# VaccImm: simulating peptide vaccination in cancer therapy

**DOI:** 10.1186/1471-2105-14-127

**Published:** 2013-04-15

**Authors:** Joachim von Eichborn, Anna Lena Woelke, Filippo Castiglione, Robert Preissner

**Affiliations:** 1 Charité – Universitätsmedizin Berlin, Institute for Physiology, Structural Bioinformatics Group, Lindenberger Weg 80, Berlin, 13125, Germany; 2 Freie Universität Berlin, Institut für Chemie, Fabeckstrasse 36a, Berlin, 14195, Germany; 3 National Research Council of Italy, Institute for Computing Applications, Rome, Italy

**Keywords:** Systems biology, Immunoinformatics, Cancer, Modelling, Proteins, Protein interaction

## Abstract

**Background:**

Despite progress in conventional cancer therapies, cancer is still one of the leading causes of death in industrial nations. Therefore, an urgent need of progress in fighting cancer remains. A promising alternative to conventional methods is immune therapy. This relies on the fact that low-immunogenic tumours can be eradicated if an immune response against them is induced. Peptide vaccination is carried out by injecting tumour peptides into a patient to trigger a specific immune response against the tumour in its entirety. However, peptide vaccination is a highly complicated treatment and currently many factors like the optimal number of epitopes are not known precisely. Therefore, it is necessary to evaluate how certain parameters influence the therapy.

**Results:**

We present the VaccImm Server that allows users to simulate peptide vaccination in cancer therapy. It uses an agent-based model that simulates peptide vaccination by explicitly modelling the involved cells (immune system and cancer) as well as molecules (antibodies, antigens and semiochemicals). As a new feature, our model uses real amino acid sequences to represent molecular binding sites of relevant immune cells. The model is used to generate detailed statistics of the population sizes and states of the single cell types over time. This makes the VaccImm web server well suited to examine the parameter space of peptide vaccination *in silico*. VaccImm is publicly available without registration on the web at http://bioinformatics.charite.de/vaccimm; all major browsers are supported.

**Conclusions:**

The VaccImm Server provides a convenient way to analyze properties of peptide vaccination in cancer therapy. Using the server, we could gain interesting insights into peptide vaccination that reveal the complex and patient-specific nature of peptide vaccination.

## Background

To mount an immune response against tumours is the objective of immunotherapy and constitutes a promising alternative to conventional methods [1]. However, the applicability of this approach remains limited so far, because of largely undefined treatment parameters. For instance, the success of immunotherapy depends strongly on the cancer epitope sequence, the individual major histocompatibility complex (MHC) genotype of the individual, and on the overall immune response dynamics. To explore this parameter space systematically, we have developed an agent-based model simulating a certain line of immunotherapy called peptide vaccination [2]. Our model VaccImm has the ability to take the MHC genotype, the amino acid epitope sequences and the spatial cell dynamics into account.

Here, we present a server that allows users to run these simulations online. Several parameters, like the cancer antigens and the MHC genotype of the virtual individual, can be selected for the simulation.

## Implementation

Agent-based models are well suited to model the immune system. They consist of independent agents, each represented with individual properties and behavioural rules. For example, every single cell is localized within a given simulated volume, has a specific type, clonotype (in case of lymphocytes) and developmental stage. This allows agent-based models to simulate the immune system, in which the different cells and cell types may be distributed very inhomogenously [3].

Back in 1992, Celada and Seiden published a cellular automaton called ImmSim to model cellular interactions in the immune system [4]. In this model, the interactions of immune cells were rather simplistically modelled by bit string complementarity. Nevertheless, this automaton was able to reproduce basic immune properties like clonal expansion of B-cells and T-cells after stimulation as well as more advanced immune system traits like the competition between cross reacting clones [5]. Since 1992, several other rule based models have been developed in order to simulate the immune system [6,7]. The original ImmSim has been upgraded and extended in the meantime, leading to different forks like C-ImmSim [8] and the mice-specific SimTriplex [9].

C-ImmSim is a refined version of the original ImmSim model that was ported to ANSI C language. However, the interaction between immune cells is still modelled as bit strings in C-ImmSim, thus having no direct translation to biologically meaningful amino acid sequences. Therefore, Rapin et al. developed an extension of C-ImmSim simulating the branch of bacterial infection [10,11]. As immune reactions against cancer cells follow different rules, they left out this part of C-ImmSim.

In a completely independent approach, we have created VaccImm - another extension of C-ImmSim - that now simulates immune reactions against cancer using amino acid sequences and knowledge-based interaction potentials to predict cell interaction. The parameters used in VaccImm were carefully examined elsewhere [2].

### Technical background

VaccImm models a three-dimensional Cartesian lattice which contains the simulated cells (cancer cells, helper T-cells, cytotoxic T-cells, B-cells, dendritic cells, macrophages) and molecules (antigens, antibodies, interleukin-2 and a danger signal that acts as a general activator of macrophages). These cells and molecules can interact and move within the lattice according to their behavioural rules. The model simulates an adaptive immune response against cancer. The thymus selection is modelled implicitly. All T-cells are checked for reactivity against MHC and self-peptides before being introduced in the simulation. Only cells having sufficiently high reactivity against the own MHC complexes (positive selection) and sufficiently low reactivity against self-peptides (negative selection) will be able to enter the simulation.

As one input parameter for the model, a certain antigenic sequence is given. Peptides for injection originating from this protein sequence are predicted for their binding capability to MHC I or MHC II using acknowledged prediction algorithms (consensus [12] for MHC I, smm_align [13] for MHC II) from the Immune Epitope Database [14].

To assess the binding probability of a receptor-ligand pair in amino acid dependent manner, knowledge-based interaction potentials are a convenient solution. We developed different interaction potentials for B-cell receptors and T-cell receptors to be used in VaccImm and we have shown that they are able to clearly distinguish between random complexes and experimentally observed ones (details are described elsewhere [2]).

### Simulation steps

The simulation is carried out in discrete time steps, each corresponding to eight hours of real life, which corresponds to one cell division cycle. In each time step the cells can interact, move and generally follow their behavioural properties with respect to their environment.

A brief outline of the single steps taken during the simulation is:

1) Injection of peptides, either emulsified in adjuvant or not. Adjuvant is modelled using the general danger signal mentioned before.

2) Antigen presenting cells take up the peptides. In the case of macrophages and dendritic cells, phagocytosis happens in an unspecific manner whereas B-cells take up only antigens they recognize. Whether antigens are recognized by B-cells or not is computed using the B-cell interaction potentials.

3) MHC I and II processing and presentation of peptides by antigen presenting cells.

4) If a peptide presented on MHC I or MHC II is recognized by cytotoxic T-cells (TC) or helper T-cells (TH), respectively, T-cells get activated, duplicate and create memory cells. Whether recognition takes place or not is assessed using the T-cell interaction potentials.

5) Both humoral and cytotoxic immune response is initiated. Activated TCs kill the cells they recognize, thereby eliminating the tumour. Recognition is again computed using the T-cell interaction potential. B-cells that are stimulated by THs duplicate into memory cells and plasma cells. These plasma cells produce antibodies that clear the antigen.

## Results and discussion

**Figure 1:**
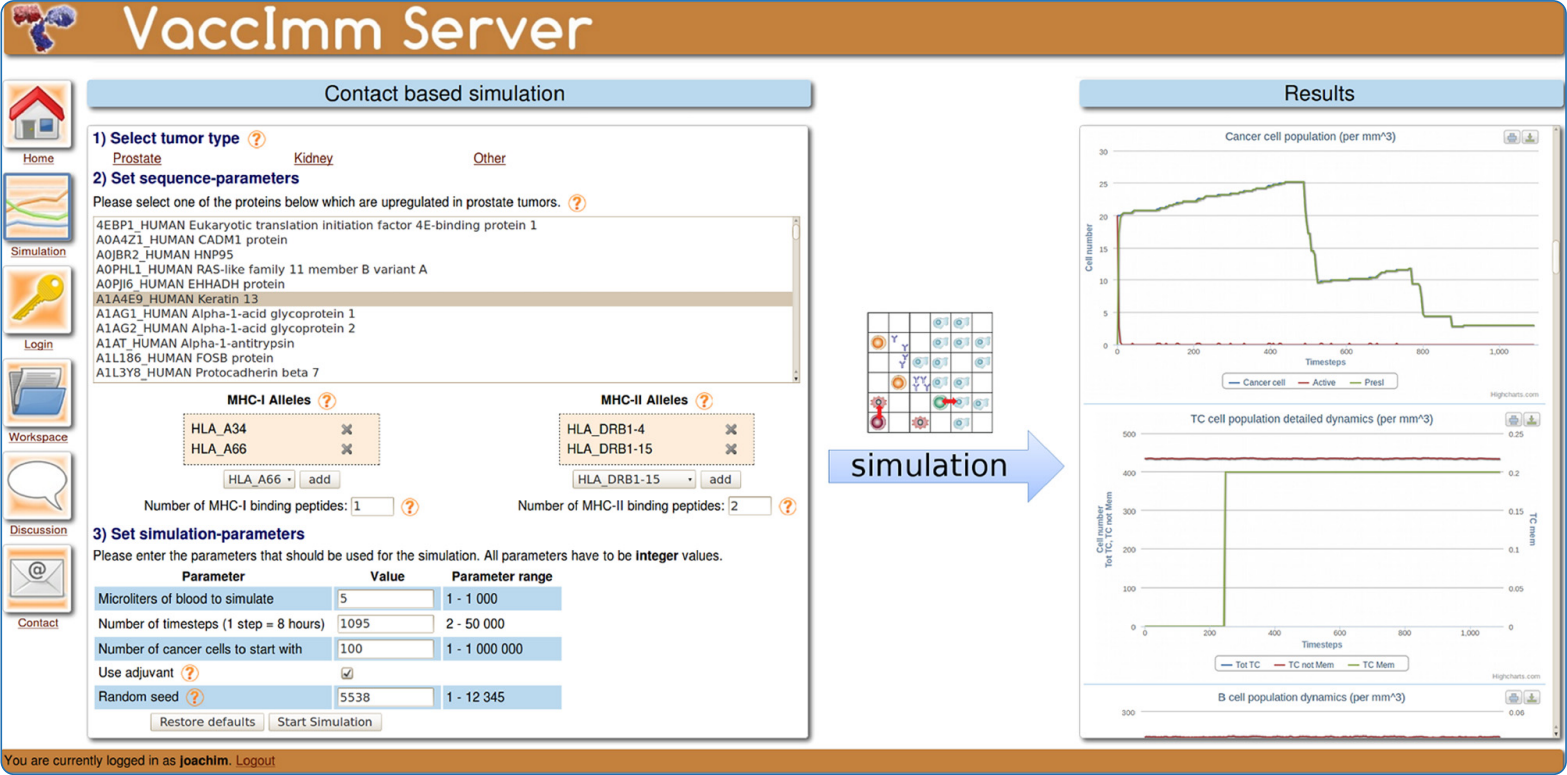
**Scheme illustrating the workflow of the VaccImm server.** On the left the input parameters are shown and on the right hand side the interactive results plots.

The simulation is freely available without registration on our server http://bioinformatics.charite.de/vaccimm. The server offers possibilities to access the results of previous performed simulations in a personal “workspace” or to perform new simulations (Figure 1). Although the server can be used without registration, the registration offers the advantage that the simulation results are protected by the account password, such that they cannot be seen or deleted by other users. It is also possible for the users to discuss their results in an integrated discussion board.

To start a new simulation, the user can decide, whether urological cancer (kidney or prostate) or another cancer type should be simulated. If one of the former types is chosen, the user can select the antigen from a list of proteins that are over-expressed in this tumour type [15]. Alternatively, a protein-sequence can be entered.

In the next step, the MHC-genotype of the virtual individual can be selected from a list of all human MHC-alleles. With this information, the strongest MHC-binding peptides are successively computed using prediction tools from the Immune Epitope Database [16]. These are the ones used for vaccination; the user can then set how many different peptides are to be injected.

Finally, some general parameters of the simulation can be changed, i.e., the simulated volume, the duration of the simulation and the initial tumour size.

The simulation results consist of the detailed dynamics of immune and cancer cell populations over time. They are presented as a number of interactive plots that enable the user to hide and show individual data lines in the plot and to get the exact cell population size at any given time. Each plot is annotated with a description that states what it is showing. Furthermore, the plots can be downloaded as image files and the raw simulation output data is accessible.

In the next sections, we will analyze how strong the influences of the above-mentioned parameters are on the outcome of the simulation.

### Dependence on the type of antigen

Clinical studies of peptide vaccination in cancer treatment usually use peptides from several different cancer proteins in the hope that the immune system will react to at least one of them [17]. The problem of finding an immunogenic epitope able to induce an effective immune response *in vivo* is one of the critical steps in immune therapy.

**Figure 2:**
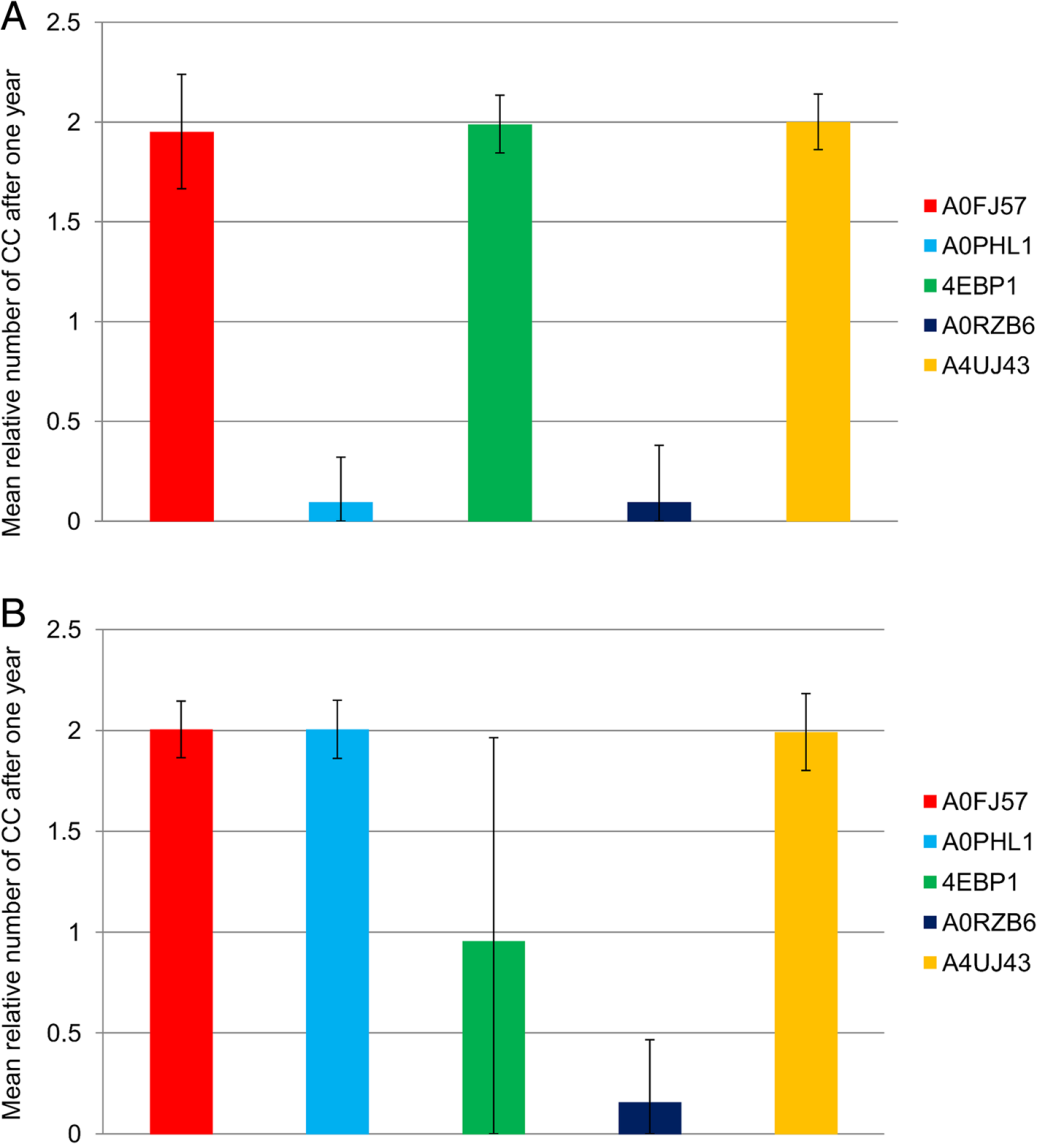
**Dependence on the type of antigen.** The relative number of cancer cells after one year of treatment compared to the tumour size at the beginning of the simulation is depicted. The mean number of cancer cells at the end of simulation is shown. Data were obtained from 500 simulations. **A**: MHC-genotype is HLA-A*01 | HLA-B*08 | HLA-DPA1/DPB1 | HLA-DRB3. **B**: MHC genotype is HLA-A*02 | HLA-B*44 | HLA-DQA1/DQB1 | HLA-DRB5. For all experiments, one hundred initial cancer cells were simulated within 5 μl of blood that were treated with injections of peptides emulgated in adjuvant starting at time point zero and being repeated five times at an interval of 28 days. Antigens were chosen from over-expression data in kidney tumours. Two MHC I-binding peptides and two MHC II-binding peptides were injected. Description of antigen UniProt-IDs: A0FJ57_HUMAN: Wilms tumour 1; A0PHL1_HUMAN: RAS-like family 11 member **B** variant **A**; 4EBP1_HUMAN: Eukaryotic translation initiation factor 4E-binding protein 1; A0RZB6_HUMAN: Endoplasmic reticulum chaperone; A4UJ43_HUMAN: Brain type mu-glutathione S-transferase.

To investigate the influence of the antigen on the success of peptide vaccination therapy, we tested five different antigens while keeping all other parameters constant (Figure 2). The relative number of cancer cells one year after starting the treatment clearly depends on the antigen. Vaccinating individuals with the MHC-genotype shown in Figure 2A with peptides derived from Wilms tumour 1 (Uni-Prot-ID: A0FJ57), the eukaryotic translation initiation factor 4E-binding protein 1 (4EBP1) or the brain type mu-glutathione S-transferase (A4UJ43), induces almost no immune response, as can be seen by the fact that the amount of cancer cells has doubled after one year in most of the simulations. In contrast, the injection of peptides from RAS-like family 11 member B variant A (A0PHL1) or the endoplasmic reticulum chaperone (A0RZB6) leads to eradication of the tumour within one year in most of the simulations. However, the individuals with the MHC-genotype shown in Figure 2B do not react against RAS-like family 11 member B variant A (A0PHL1), but exhibit an immune response against eukaryotic translation initiation factor 4E-binding protein 1 (4EBP1) in about 50% of the simulations. Therefore, the MHC-genotype seems to influence the success of treatment dramatically (see next section). The large qualitative differences in reactivity against the different antigens ranging from no response to a complete response are observed in classical vaccination approaches as well [18]. Different antigens might produce peptides having very different MHC-binding ability or being simply more or less immunogenic towards the immune system. Therefore, a tailored patient-specific choice of antigen in the vaccination therapy is of great importance, as it determines which peptide sequences can be possibly derived from it.

### Dependence on the MHC-genotype

The peptide binding characteristics of MHC alleles differ significantly and susceptibility to several diseases has been associated with the MHC-genotype [19,20]. Therefore, we wanted to investigate the influence of the MHC-genotype on the success of peptide vaccination therapy.

**Figure 3:**
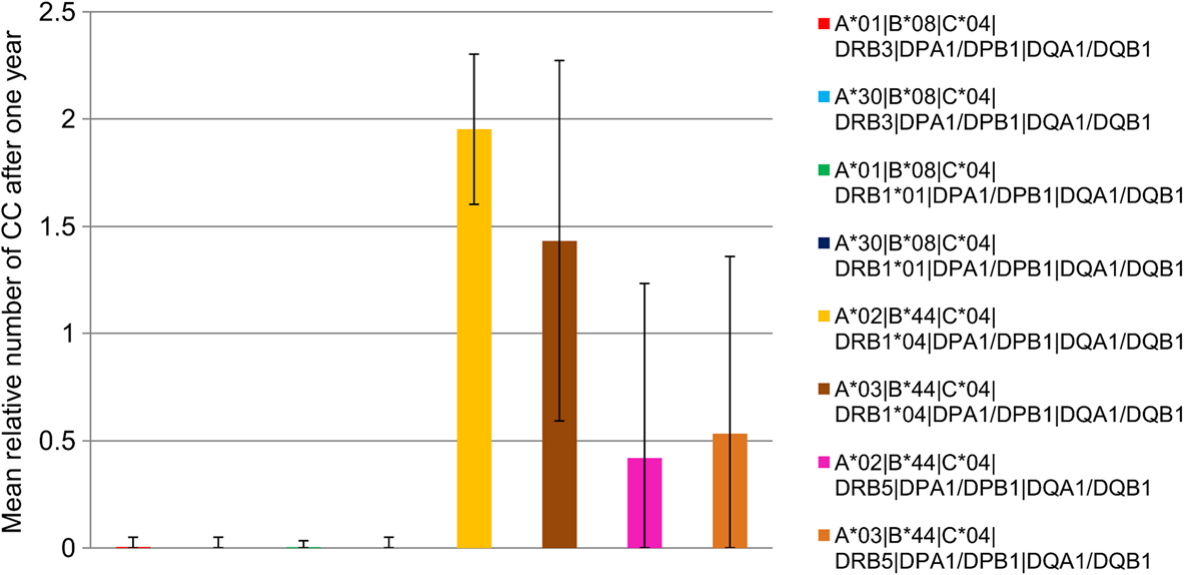
**Dependence on the MHC-genotype.** The relative number of cancer cells after one year of treatment compared to the tumour size at the beginning of the simulation is depicted. The mean number of cancer cells at the end of simulation is shown. Data were obtained from 500 simulations. MHC-genotypes are given in the legend of the graph. For all experiments, one hundred initial cancer cells were simulated within 5 μl of blood that were treated with injections of peptides emulgated in adjuvant starting at time point zero and being repeated five times at an interval of 28 days. The antigen 7B2_HUMAN (Neuroendocrine protein 7B2) was chosen from over-expression data in kidney tumours. Two MHC I-binding peptides and two MHC II-binding peptides were injected.

For this purpose, we compared simulations performed with eight different MHC-genotypes, keeping all other parameters constant (Figure 3). In the first four MHC-allele combinations, the immune response was very strong and able to eradicate the tumour completely in almost all of the simulations. In contrast, in the last four MHC-allele combinations the immune system exhibited a weaker response and full eradication occurred only in some cases. Still, there are significant differences within the last four MHC-allele combinations. In both experiments including HLA-DRB1*04 (Figure 3 yellow and brown bars), the immune response was absent in almost all of the simulations, while the tumour is eradicated in the majority of the simulations including HLA-DRB5 (Figure 3 black and pink bars). Interestingly, simulations with HLA-A*02 in combination with HLA-DRB5 exhibit a stronger immune response than with HLA-A*03/HLA-DRB5, while the immune response is similar for HLA-A*02 and HLA-A*03 if combined with HLA-DRB1*04.

This demonstrates, once more, the complex nature of the immune response against tumours. Injecting peptides from the same antigen might induce a strong immune response having a certain MHC-allele combination, while the response could be completely absent when having a different allele combination, as can be seen in Figure 2. It is worth emphasizing that in VaccImm, the injected peptides are chosen in accordance with the MHC alleles. Therefore, a possible explanation for the different responses to the same antigen presented on different MHC-alleles could be that the peptide sequences binding to one MHC are different from the peptides binding to other MHCs, which results in a difference in immunogenicity of these different peptides.

This experiment is an indication that peptide vaccination remains a personalized treatment because injecting the peptides derived from the same antigen might have a completely different outcome depending on the individual MHC-alleles present.

### Dependence on the number of injected peptides

When a possible cancer target is found by expression analysis, the respective peptide presented on MHC I or MHC II is often unknown. Consequently, prediction algorithms are usually used to find cancer epitopes presented on MHCs. Likewise, we have used this kind of algorithm, bearing in mind that they are of limited accuracy [21] and the highest ranked peptide could turn out not to be the one eventually presented by APCs. Therefore, we investigated whether injecting more peptides from the same antigen increases the chance of successful treatment.

**Figure 4:**
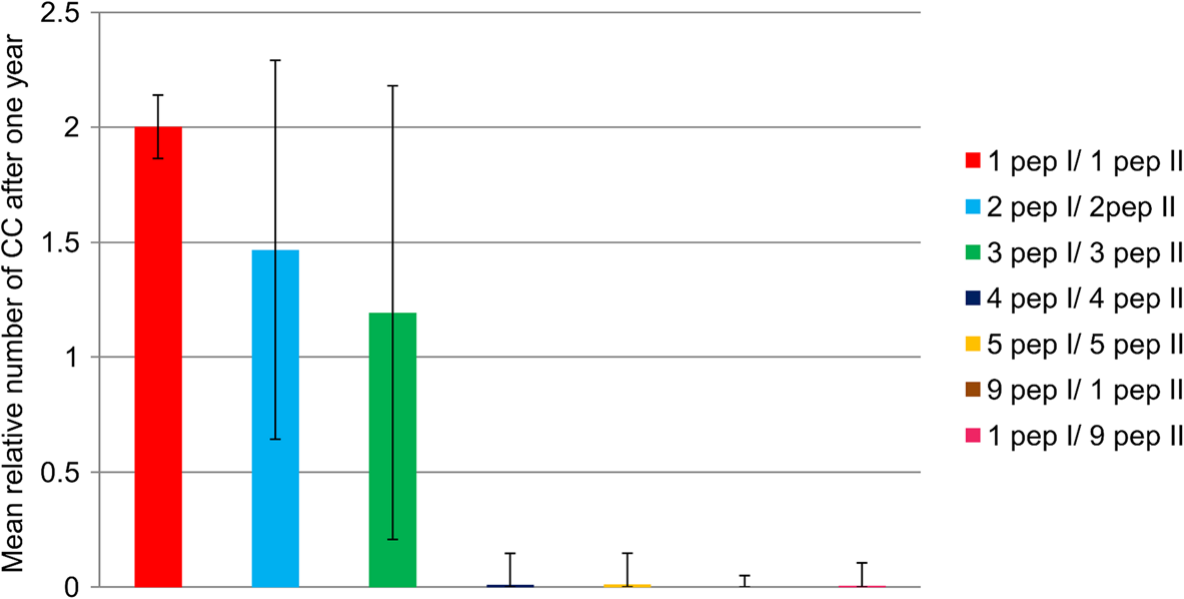
**Dependence on the number of peptides injected.** The relative number of cancer cells after one year of treatment compared to the tumour size at the beginning of the simulation is depicted. The mean number of cancer cells at the end of simulation is shown. Data were obtained from 500 simulations. Number of peptides for each type of MHC is given in the legend, e.g. 1 pep I: one peptide binding to MHC I. The MHC-genotype is HLA-A*03 | HLA-B*44 | HLA-C*04 | HLA-DRB1*04 | HLA-DQA1/DQB1 | HLA-DPA1/DPB1. For all experiments one hundred initial cancer cells were simulated within 5 μl of blood that were treated with injections of peptides emulgated in adjuvant starting at time point zero and being repeated five times at an interval of 28 days. The antigen 7B2_HUMAN (Neuroendocrine protein 7B2) was chosen from over-expression data in kidney tumours.

For that purpose, we compared simulations with different numbers of peptides from the same antigen while keeping all other parameters constant (Figure 4). The immune response to this antigen differs clearly depending on the number of peptides injected. When injecting one peptide for each MHC type, almost no immune response is observed, whereas the tumour is eradicated in almost all of the simulations where four or more peptides for each MHC type are injected. Interestingly, the likelihood of inducing an immune response increases stepwise with the number of peptides. This observation indicates that T-cells not only become activated against the most immunogenic peptide. Instead, different T-cell clones expand whilst reacting against several of the injected peptides and have an additive effect, resulting in the stronger immune response.

It seems that a strong responsiveness of TCs can complement a weaker TH responsiveness and vice versa; for example, injecting one MHC II and nine MHC I peptides or one MHC I and nine MHC II peptides induces an equally strong immune response whereas injecting one peptide for both MHC types does not. This observation highlights the interconnection of the immune cells. TCs need the help of THs to become activated, while their reactivity depends on the immunogenicity and binding properties of MHC I peptides, but also on the reactivity of THs. In some cases, the reactivity of TCs and THs may complement each other, although the actual mechanisms behind are more complex than simple dose-dependent reactions.

It should be mentioned that this dependency on the number of injected peptides is not observed for all antigens and MHC-allele combinations. Some antigens fail to induce an immune response regardless of the number of injected peptides whereas others lead to tumour eradication even when only one peptide for each MHC type is injected (data not shown).

The observation above suggests using as many peptides as possible in planning a clinical study. However, this is not practically because every injected peptide bears the risk of inducing an autoimmune disease. What must be carefully taken into account is which peptides are likely to induce a strong favoured response against the tumour but a low or absent response against self proteins.

### Dependence on the initial tumour size

Cancer dormancy describes the phenomenon that small tumours can be kept in check without treatment for a longer period of time [22]; in contrast, larger tumours often grow exponentially. In general, the chosen therapy depends on the tumour size. For example, for a prostate tumour of a considerable size, radical prostatectomy is often the treatment of choice, whereas smaller tumours are frequently kept under active surveillance for a long time [23]. It is therefore of special interest to analyze how the initial tumour size influences the success rate of the immune therapy.

**Figure 5:**
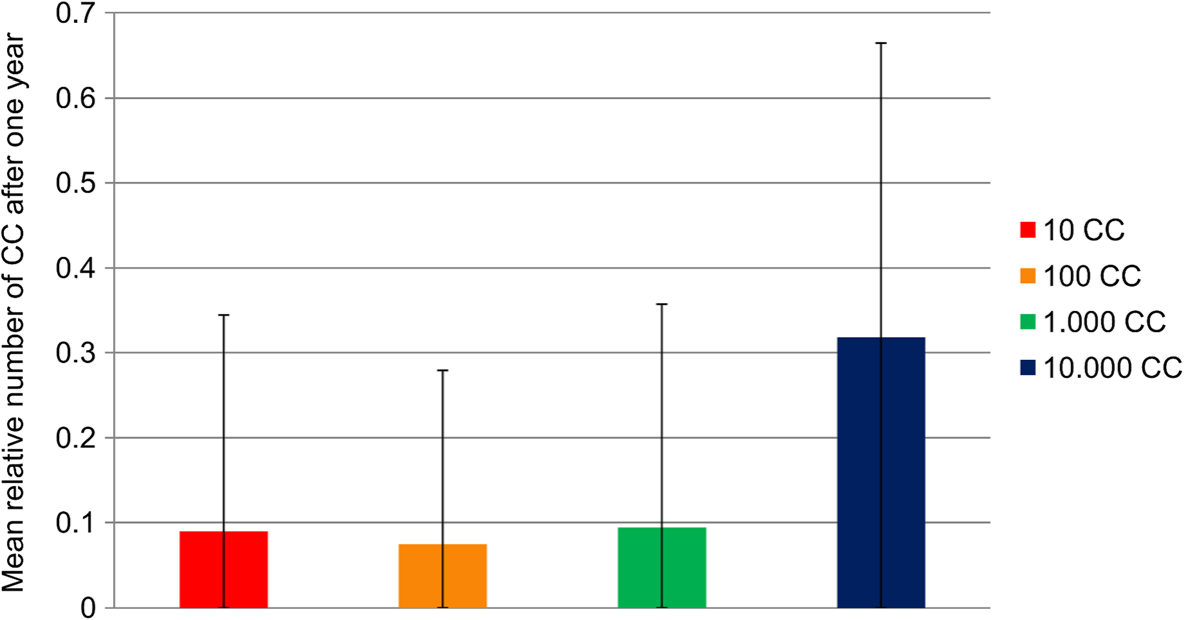
**Dependence on the initial number of cancer cells.** The relative number of cancer cells after one year of treatment compared to the tumour size at the beginning of the simulation is depicted. The mean number of cancer cells at the end of simulation is shown. Data were obtained from 1,000 simulations. The number of cancer cells at the beginning of the simulation is given in the legend. MHC-genotype is HLA-A*01 | HLA-B*08 | HLA-DRB3 | HLA-DPA1/DPB1. For all experiments, simulation space was 5 μl of blood and injections of peptides emulgated in adjuvant started at time point zero being repeated five times at an interval of 28 days. The antigen A0PHL1_HUMAN (RAS-like family 11 member B variant A) was chosen from over-expression data in prostate tumours. Two MHC I-binding peptides and two MHC II-binding peptides were injected.

For a given parameter set, simulations starting with 10, 10^2^ or 10^3^ cancer cells have almost the same outcome with a mean tumour size of below 10% after one year whereas the size increases to over 30% when starting with 10^4^ cancer cells (Figure 5). Hence, the initial number of cancer cells when starting the treatment has an impact on the outcome. In contrast to the parameters discussed previously, it seems that the initial number of cancer cells has only a quantitative but not a qualitative influence.

The results in Figure 5 indicate that if the immune system is activated sufficiently against a tumour, eradication is only a matter of time. Yet, the problem with cancer cells is that they are usually composed of an ever growing diversity of cells with different expression patterns; a fact that is not considered in our model so far. Thus, a larger tumour has more time to mutate or to change the expression pattern and hence a cure with peptide vaccination is less likely to have a positive effect.

Our future plan is to include the diversity of cells, since without this parameter we cannot deduce from the results of this experiment whether the initial tumour size is an indication of success for peptide vaccination therapy.

## Conclusions

We have presented VaccImm, a user-friendly server to simulate the effect of peptide vaccination in cancer therapy. We believe this tool is very useful for analyzing parameters of peptide vaccination in cancer therapy. It is the first web server that can model cancer immunotherapy based on cancer epitope sequences and MHC genotypes. All sequence-related parameters can be selected by the user, along with parameters concerning size and duration of the simulation. The output shows detailed cell population statistics for all modelled cell types. Their population sizes and activation states can be studied over time.

As stated in the introduction, VaccImm can be very useful to explore the parameter space of peptide vaccination. For instance, we have performed some studies analyzing the influence of several parameters on the success of peptide vaccination. The results presented above revealed the complex nature of peptide vaccination as the influences of the single parameters strongly depend upon each other. The selection of the antigen is crucial for peptide vaccination, because the antigen must be able to evoke an immune response. However, the quality of a potential antigen in turn depends on the MHC-genotype of the patient. This is because the antigens are processed and subsequently different MHCs may present different parts of the antigens. VaccImm accounts for this by predicting the MHC binding peptides based on the MHC-genotype. It could also be shown, that a successful therapy gets more likely the more different peptides are injected, still bearing the risk of an autoimmune reaction. One feature that is currently not included in our model is mutation of cancer cells. Therefore, the initial size of the tumour has no qualitative effect on the outcome of the simulation. If mutation of tumour cells was possible, one would expect a higher probability for a cell in a large tumour to mutate in such a way that it is not or less affected by the evoked immune response. Thereby the probability of a successful treatment can be expected to become smaller with increasing tumour size. Due to the modular structure of our model, it is easily possible to add such behaviour in a future version.

## Availability and requirements

**Project name:** VaccImm

**Project home page:**http://bioinformatics.charite.de/vaccimm/

**Operating systems:** Platform independent

**Programming language:** C and PHP

**Other requirements:** Up-to-date web browser with JavaScript enabled

**License:** VaccImm is available free of charge without registration

**Any restrictions to use by non-academics:** None

## Abbreviations

MHC: Major histocompatibility complex; TC: Cytotoxic T-cell; TH: Helper T-cell.

## Competing interests

The authors declare that they have no competing interests.

## Authors’ contributions

JvE implemented the web server and helped with the implementation of the amino-acid based molecular recognition. AW implemented the amino-acid sequence based recognition. FC is the developer of the original C-ImmSim code and discussed critical aspects of implementing the amino-acid sequence based recognition. RP discussed the concept of the project and supervised it. All authors read and approved the final manuscript.
